# MicroRNA-181c Exacerbates Brain Injury in Acute Ischemic Stroke

**DOI:** 10.14336/AD.2016.0320

**Published:** 2016-12-01

**Authors:** Qingfeng Ma, Haiping Zhao, Zhen Tao, Rongliang Wang, Ping Liu, Ziping Han, Shubei Ma, Yumin Luo, Jianping Jia

**Affiliations:** ^1^Department of Neurology and Cerebrovascular Diseases Research Institute, Xuanwu Hospital of Capital Medical University; ^2^Neurodegenerative Laboratory of Ministry of Education of the People’s Republic of China; ^3^Beijing Institute for Brain Disorders; ^4^Beijing Key Laboratory of Translational Medicine for Cerebrovascular Disease, Beijing, China

**Keywords:** microRNA-181, stroke, microglia, neuron, apoptosis

## Abstract

MicroRNA-181 (miR-181) is highly expressed in the brain, and downregulated in miRNA expression profiles of acute ischemic stroke patients. However, the roles of miR-181c in stroke are not known. The clinical relevance of miR-181c in acute stroke patients was evaluated by real-time PCR and correlation analyses. Proliferation and apoptosis of BV2 microglial cells and Neuro-2a cells cultured separately or together under oxidative stress or inflammation were assessed with the Cell Counting Kit-8 and by flow cytometry, respectively. Cerebral ischemia was induced by middle cerebral artery occlusion (MCAO) in C57/BL6 mice, and cerebral infarct volume, microglia activation, and expression of pro-apoptotic factors were evaluated by 2,3,5-triphenyl-2H-tetrazolium chloride staining, immunocytochemistry, and western blotting, respectively. Plasma levels of miR-181c were decreased in stroke patients relative to healthy individuals, and were positively correlated with neutrophil number and blood platelet count and negatively correlated with lymphocyte number. Lipopolysaccharide (LPS)/hydrogen peroxide (H_2_O_2_) treatment inhibited BV2 microglia proliferation without inducing apoptosis, while miR-181c reduced proliferation but increased the apoptosis of these cells with or without LPS/H_2_O_2_ treatment. LPS/H_2_O_2_ induced apoptosis in Neuro-2a cells co-cultured with BV2 cells, an effect that was potentiated by miR-181c. In the MCAO model, miR-181c agomir modestly increased infarct volume, markedly decreased microglia activation and B cell lymphoma-2 expression, and increased the levels of pro-apoptotic proteins in the ischemic brain. Our data indicate that miR-181c contributes to brain injury in acute ischemic stroke by promoting apoptosis of microglia and neurons via modulation of pro- and anti-apoptotic proteins.

Stroke-related disability represents a significant healthcare burden worldwide [1]. As such, more markers for the early diagnosis of stroke and prediction of patient prognosis are needed [2]. Inflammation and oxidative stress are major factors that contribute to the pathophysiology of stroke; cytokines and reactive oxygen species are produced upon activation of microglia, which accumulate around degenerating neurons following stroke and assume a neurotoxic or neuroprotective phenotype [3]. Activated microglia rescue neurons by phagocytosis of cytotoxins and cellular debris and by producing anti-oxidant enzymes [3].

MicroRNAs (miRNAs) are involved in post-transcriptional regulation of genes and are potential biomarkers for a variety of cellular processes and diseases [4]. However, the fact that a single miRNA can have multiple targets makes it difficult to predict the function of miRNAs in stroke. The miR-181 family is evolutionarily conserved and highly expressed in the brain [5], and has been implicated in apoptosis and inflammation [6-11]. MiR-181-a/b/c is downregulated in the rat brain after transient focal ischemia [12]; similarly, miR-181-a/b/c/d levels in mice were decreased in the ischemic penumbra and increased in the ischemic core after transient focal ischemia [13]. MiR-181a was shown to exacerbate injury in a mouse stroke model [13], and induced Neuro-2a cell death following serum deprivation and oxidative stress [14]. MiR-181a also induced mitochondrial dysfunction and increased cell death in primary astrocytes via modulation of B cell lymphoma (Bcl)-2 family members [9,13]. In contrast, miR-181c protected neurons grown in BV2-conditioned medium under oxygen-glucose deprivation by suppressing the expression of Toll-like receptor 4 and downstream cytokines [15,16]. However, the precise function of miR-181c in ischemic stroke is unknown.

MiRNA expression profiling of peripheral lymphocytes from acute ischemic stroke patients revealed that the three miR-181 family members namely, hsa-miR-181a/c/d, were among the top 22 that were downregulated, implying that this family is clinically relevant. MiR-181c showed the greatest change among these family members; therefore, this present study investigated the involvement of miR-181c in acute ischemic stroke by culturing microglia and neuronal cells alone or together under conditions of oxidative stress and inflammation, and using a mouse model of stroke.

## MATERIALS AND METHODS

### Clinical sample collection

The use of the human blood samples for research purposes was approved by the Ethics Committee of Capital Medical University. Informed, written consent was obtained from all participants. Criteria for inclusion of subjects have been previously described [17]. Inclusion criteria were as follows: (1) diagnosis of first ischemic stroke based on clinical information and magnetic resonance imaging; (2) male patients aged 55-65 years old; (3) presentation of subjects within 72 h of the event; (4) National Institutes of Health Stroke Scale (NIHSS) score between 4 and 15; and (5) Trial of ORG 10172 in Acute Stroke Treatment stroke subtype of large-artery atherosclerosis. Age- and sex-matched non-stroke control subjects were randomly selected based on results of physical examination. Lymphocyte miRNA profiles were analyzed in three acute stroke patients and three control subjects. Lymphocytes were isolated using lymphocyte separation medium (TBD Biological Technology Development Center, Tianjin, China) and were analyzed with a High Density Human MiRNA array (v.3 release 12) containing 851 human miRNAs (Agilent Technologies, Santa Clara, CA, USA). The random variance model with a corrected t test was used to filter genes that were differentially expressed in healthy controls and stroke patients. After significance and false positive rate analyses, differentially expressed genes were selected based on the threshold P value.

### Real-time PCR (RT-PCR) quantification of miR-181c/d levels

Blood samples were collected in heparinized tubes and centrifuged at 2000 ×*g* for 10 min. Plasma miR-181c and -181d levels of stroke patients and healthy subjects were detected by RT-PCR. Total RNA was isolated by adding 1 ml TRIzol reagent (Invitrogen, Carlsbad, CA, USA) to 300 μl plasma; 50 ng of RNA were reverse transcribed using SuperScript III Reverse Transcriptase (Invitrogen) with miRNA RT primer (GenePharma, Shanghai, China). The expression of mature human miRNAs was determined by a stem-loop RT-PCR system using Maxima SYBR Green qPCR Master Mix (Fermentas, Burlington, Canada) and a StepOne sequence detector (Applied Biosystems, Foster City, CA, USA). MiRNA levels were normalized to that of U6 and were calculated as the inverse log of the cycle threshold to determine the relative fold change in miRNA gene expression level. Forward and reverse primers used for RT-PCR were as follows: hsa-miR-181c: 5’-TTCTTCAACATTCAACC TGTCG-3’ and 5’-TATCGTTGTACTCCAGACCA AGAC-3’; hsa-miR-181d: 5’-CTCATAAACATTCATT GTTGTC GG-3’ and 5’-CTCATAAACATTCAT TGTTGTCGG-3’; U6: 5’-ATTGGAACGATACAGAG AAGATT-3’ and 5’-GGAACGCTTCACGAATTTG-3’.

### Cell culture and miR-181c agomir transfection

Murine BV2 and Neuro-2a cells were cultured in Dulbecco’s Modified Eagle’s Medium supplemented with 10% (v/v) heat-inactivated fetal bovine serum, 100 U/ml penicillin, and 100 mg/ml streptomycin at 37°C in 5% CO_2_/95% air. Sense and antisense miR-181c agomirs were synthesized by GenePharma based on the following sequences: has-miR-181c, 5'-AACAUUCAACCUGUC GGUGAGU-3' and 5'-UCACCGACAGGUUGAAUG UUUU-3'; and scrambled miRNA (negative control), 5'-UUCUCCGAACGUGUCACGUTT-3' and 5'-ACG UGACACGUUCGGAGAATT-3'. Neuro-2a cells were transfected with miR-181c agomir at a final concentration of 50 nmol/l using Lipofectamine RNAiMAX Transfection Reagent (Invitrogen) according to the manufacturer’s protocol, and 24 h later, the cells were treated with H_2_O_2_ (200 μM) or lipopolysaccharide (LPS; 100 ng/ml) to induce oxidative stress and inflammation, respectively, for 24 h.

BV2 microglia and Neuro-2a cells were co-cultured using transwell cell culture inserts (Dow Corning, Corning, NY, USA). Briefly, BV2 cells were seeded on the microporous membrane of transwell cell culture inserts and Neuro-2a cells were grown in the plate. All cells were transfected with miR-181c agomir and concurrently treated with H_2_O_2_ or LPS, and Neuro-2a cells and medium were analyzed 24 h later. Cell proliferation was assessed with Cell Counting Kit-8 (Dojindo Laboratories, Kumamoto, Japan) according to the manufacturer’s instructions.

### Flow cytometry analysis of apoptosiss

Neuro-2a and BV2 cells transfected for 24 h with miR-181c agomir followed by H_2_O_2_ (200 μM) or LPS (100 ng/ml) treatment for 24 h were washed twice with phosphate-buffered saline (PBS), and the apoptotic fraction was detected by staining with fluorescein isothiocyanate-conjugated annexin-V and propidium iodide (PI) using the Annexin-V/PI Apoptosis Detection kit (BD Biosciences, Franklin Lakes, NJ, USA) followed by cell sorting using a FACS Canto instrument (BD Biosciences). Annexin-V^+^/PI^-^ and Annexin-V^+^/PI^+^ cells were taken as early apoptotic and necrotic cells, respectively.

### Mouse model of focal cerebral ischemia

Animal experiments were performed in accordance with the guidelines of the Institutional Animal Care and Use Committee of Capital Medical University. Male C57BL/6J mice weighing 20-25 g were purchased from Vital River Laboratory Animal Technology Co. (Beijing, China). After intracerebroventricular injection of control or miR-181c agomir or a negative control, focal ischemia was induced by middle cerebral artery occlusion (MCAO). MiR-181c mimic (100 μmol/l) or the control (100 μmol/l) was mixed with siRNA-MATE (GenePharma), followed by incubation at room temperature for 20 min and intracerebroventricular injection (7 μl for 20 min) after 10 min. Ischemia was confirmed by monitoring regional cerebral blood flow with a laser Doppler flowmeter (PeriFlux System 5000; Perimed, Järfälla, Sweden) at a position 0.5 mm anterior and 5.0 mm lateral to bregma. Body temperature was maintained at 37.0°C during and after surgery using a temperature-controlled heating pad (CMA 150; Carnegie Medicine, Stockholm, Sweden). Mice were sacrificed 24 h after reperfusion, and their brains were quickly removed and processed for 2,3,5-triphenyl-2H-tetrazolium chloride (TTC) staining and calculation of cerebral infarct volume [18]. Six mice from each group (except for the sham group) were used for TTC staining; the remaining three mice in each group were used for western blotting and immunocytochemistry.

### Immunocytochemistry

Mice were euthanized 24 h after reperfusion by intraperitoneal injection of chloral hydrate (300 mg/kg) and were transcardially perfused with 4% w/v paraformaldehyde in PBS. After incubation for 2 h in blocking solution (0.3% Triton X-100, 2% normal goat serum, 1% bovine serum albumin, and 5% non-fat dry milk in PBS), frozen coronal sections were incubated with the primary antibodies against ionized calcium-binding adapter molecule (Iba)-1 (Wako Pure Chemical Industries, Osaka, Japan) and Bcl-2 (1:50; Cell Signaling Technology, Danvers, MA, USA). After washing with PBS, sections were incubated in a mixture of fluorescent secondary antibodies (Alexa 488-/Alexa 594-conjugated anti-mouse/-rabbit IgG). Images were acquired with an epi?uorescence microscope.

### Western blot analysis

Ipsilateral cortices were collected 24 h after ischemia and processed for western blotting as previously described [19]. Antibodies against the following proteins were used at 1:1000 dilution: activated caspase-3 and Bcl-2-associated X protein (Bax) (both from Abcam, Cambridge, UK), and β-actin (Santa Cruz Biotechnology, Santa Cruz, CA, USA). Protein bands were detected using horseradish peroxidase-conjugated secondary antibody (1:2000; Santa Cruz Biotechnology) and by enhanced chemiluminescence.

### Statistical analysis

Data were analyzed using SPSS v.11.5 software package for Windows (SPSS Inc., Chicago, IL, USA). Data are expressed in the form of mean ± standard deviation (SD), and differences between groups were evaluated by analysis of variance, the Student’s *t* test, and Kendall correlation analysis. Statistical significance was set at *P* < 0.05.

## RESULTS

### Plasma hsa-miR-181c levels are downregulated in stroke patients

**Figure 1.**
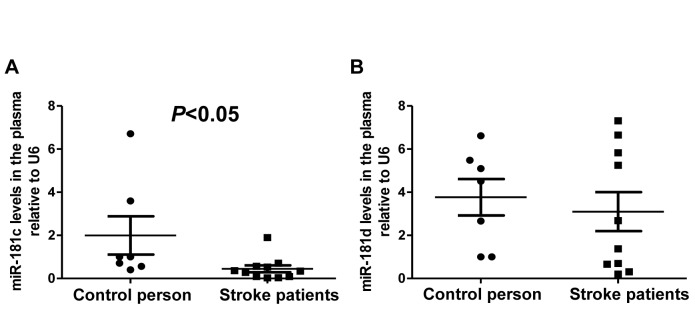
**Expression of miR-181c and miR181d in the plasma of acute stroke patients**. Plasma levels of miR-181c (A) and miR-181d (B) in acute stroke patients and controls, as determined by semi-quantitative RT-PCR (n = 7 in the control group, n = 10 in the stroke group). U6 was used to normalize expression levels of target miRNAs in different samples.

The lymphocyte miRNA microarray analysis of acute stroke patients revealed that miR-181 family members, including hsa-miR-181a/c/d, were among the top 44 down- or up-regulated miRNAs (Table 1, *P* < 0.05), implying that this family is clinically relevant. MiR-181c showed the greatest change among its members. Furthermore, RT-PCR analysis revealed that hsa-miR-181c levels were decreased in the plasma of stroke patients as compared to healthy subjects (Fig. 1A, *P* < 0.05), consistent with the change in miR-181c level obtained by the miRNA array. Hsa-miR-181d expression was also reduced in the plasma of stroke patients relative to controls, but the difference was not statistically significant (Fig. 1B). These results indicate that although miR-181c and -181d belong to the same family, their expression levels are differentially altered in response to stroke.

**Table 1:** MiRNAs down- or upregulated in lymphocytes within 72 h of acute stroke.

MiRNA	Fold change	Regulation	MiRNA	Fold change	Regulation
hsa-miR-144	-268	down	hsa-miR-572	66	up
hsa-miR-340	-233	down	hsa-miR-212	22	up
hsa-miR-152	-67	down	hsa-miR-933	19	up
hsa-miR-590-5p	-63	down	hsa-miR-494	12	up
hsa-miR-551b	-46	down	hsa-miR-638	6	up
hsa-miR-30a	-43	down	hsa-miR-133b	6	up
hsa-miR-20a*	-43	down	hsa-miR-1275	5	up
hsa-miR-193a-3p	-37	down	hsa-miR-1268	4	up
hsa-miR-330-3p	-35	down	hsa-miR-1915	4	up
hsa-miR-126*	-21	down	hsa-miR-29b-1*	4	up
hsa-miR-495	-19	down	hsa-miR-1225-5p	3	up
hsa-miR-301b	-19	down	hsa-miR-296-5p	3	up
hsa-miR-582-5p	-15	down	hsa-miR-574-5p	3	up
hsa-miR-301a	-13	down	hsa-miR-1234	2	up
hsa-miR-424	-12	down	hsa-miR-139-5p	2	up
hsa-miR-532-3p	-11	down	hsa-miR-1249	2	up
hsa-miR-99b	-10	down	hsa-miR-940	2	up
hsa-miR-181c*	-**16**	**down**	hsa-miR-1202	2	up
**hsa-miR-181c**	-**13**	**down**	hsa-miR-1281	2	up
hsa-miR-181a-2*	-**13**	**down**	hsa-miR-150	2	up
hsa-miR-181a*	-**3**	**down**	hsa-miR-33b*	2	up
**hsa-miR-181d**	-**3**	**down**	hsa-miR-1825	2	up

The asterisk (*) denotes the presence of the ‘passenger’ strand that is represented at lower levels in the steady state.

### MiR-181c levels are correlated with clinical parameters of stroke

**Figure 2.**
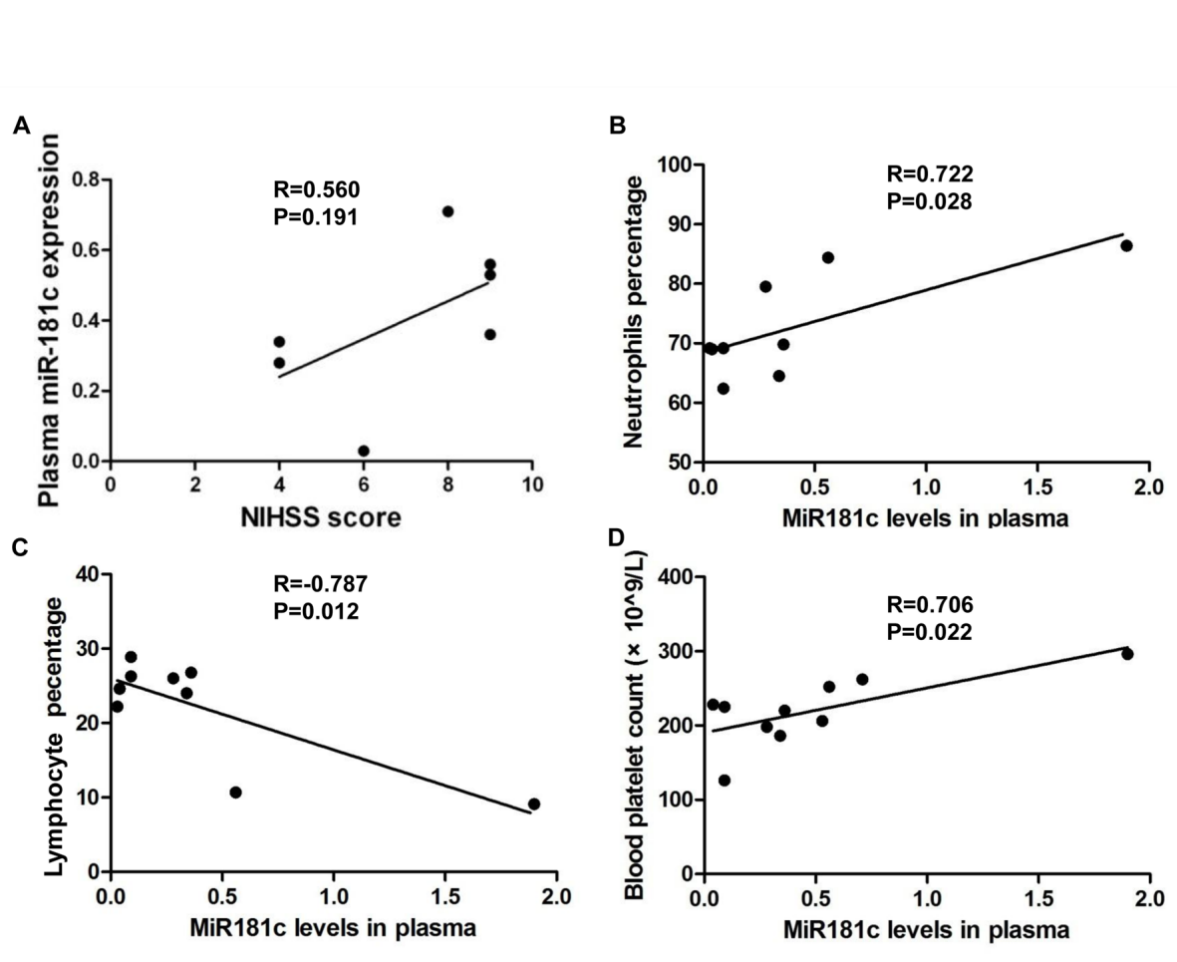
**Correlation between miR-181c level in plasma and NIHSS score (n = 7)**. (**A**), neutrophil number (n = 8) (**B**), lymphocyte number (n = 9) (**C**), and blood platelet count (n = 10) (**D**). *P* < 0.05 was used as a cutoff value.

Given that acute stroke patients had lower levels of circulating miR-181c, we examined the correlation between this and the NIHSS score, which indicated the severity of ischemic stroke. NIHSS scores were positively correlated with miR-181c levels, suggesting a neuroprotective role for miR-181c in acute ischemic stroke, even when correlation was not statistically significant (Fig. 2A). We also analyzed the correlation between miR-181c level and serum biochemical parameters. Plasma miR-181c concentration was positively correlated with neutrophil number (Fig. 2B, *P* < 0.05) and blood platelet count (Fig. 2C, *P* < 0.05), and negatively correlated with lymphocyte number (Fig. 2D, *P* < 0.05). However, plasma miR-181c level was not correlated with other risk factors such as homocysteic acid, C reactive protein, triglyceride, total cholesterol, low density lipoprotein, and ApoB levels. The positive correlation between the NIHSS score and miR-181c level suggest that miR-181c is a potential risk factor for stroke, and that miR-181c is a potential regulator of neutrophil and lymphocyte numbers in acute ischemic stroke.

### MiR-181c agomir induces apoptosis of BV2 microglial and Neuro-2a neuronal cells

**Figure 3.**
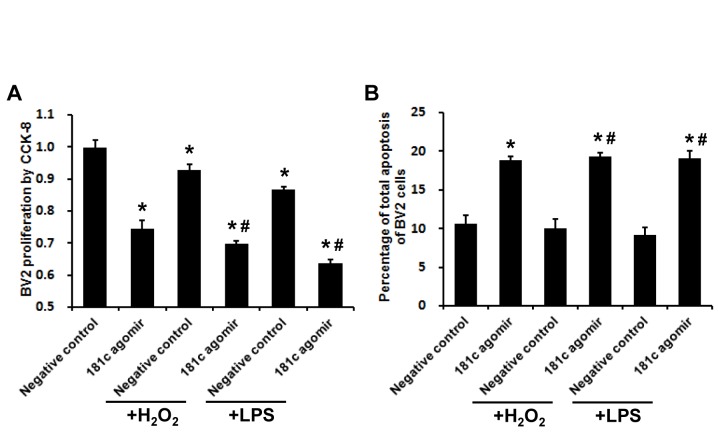
**MiR-181c agomir inhibits proliferation and induces apoptosis of BV2 microglial cells upon oxidative stress and inflammation. A**) BV2 cell proliferation was assessed with Cell Counting Kit-8. **B**) BV2 cell apoptosis was determined by flow cytometry. Values represent mean ± SEM. *P < 0.05 vs. negative control group; ^#^*P* < 0.05 vs. negative control + H_2_O_2_ or negative control + LPS group.

Given the correlation between miR-181c level and immune cell numbers in acute stroke patients, we examined the role of miR-181c in BV2 microglial cell proliferation and apoptosis under oxidative stress and inflammation. Treatment with H_2_O_2_ or LPS inhibited BV2 cell proliferation without inducing apoptosis, while treatment with miR-181c agomir suppressed proliferation and increased apoptosis in the presence or absence of H_2_O_2_/LPS treatment (Fig. 3A, B; *P* < 0.05).

**Figure 4.**
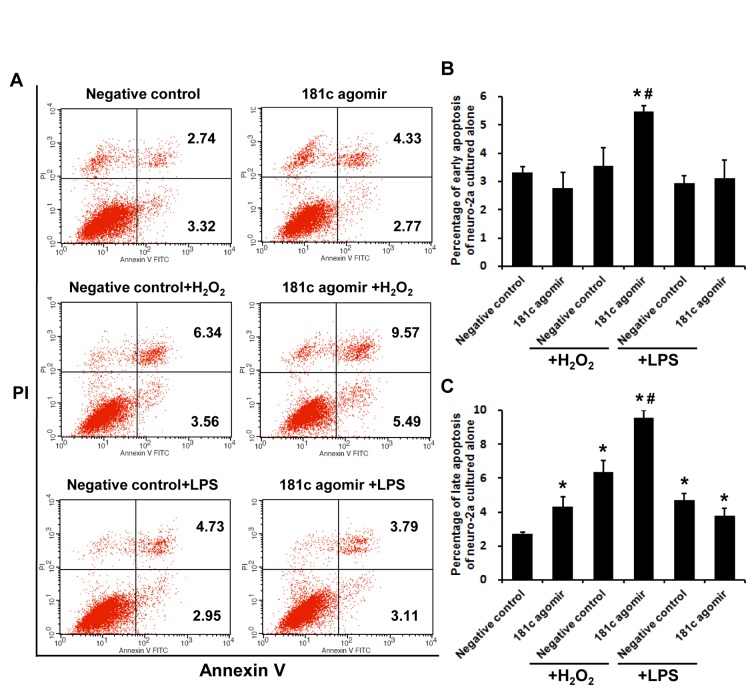
**MiR-181c agomir promotes apoptosis of Neuro-2a cells upon oxidative stress and inflammation. A**) Apoptotic cells were detected by flow cytometry. Second and fourth quadrants represent late and early apoptotic fractions, respectively. **B**) Early apoptosis of Neuro-2a cells. **C**) Late apoptosis of Neuro-2a cells. Values represent mean ± SEM. *P < 0.05 vs. negative control group; ^#^*P* < 0.05 vs. negative control + H_2_O_2_ or negative control + LPS group.

To evaluate the role of miR-181c in neurons under conditions of oxidative stress and inflammation, apoptosis of Neuro-2a cells transfected with control or miR-181c agomir followed by H_2_O_2_ or LPS treatment was assessed by flow cytometry (Fig. 4A). After 24 h of exposure to H_2_O_2_ or LPS, the late apoptotic cell fraction was increased. Treatment with miR-181c agomir along with H_2_O_2_, but not LPS, markedly increased early and late apoptosis fractions (Fig. 4B, C; *P* < 0.05). These results demonstrate that miR-181c exacerbates Neuro-2a cell apoptosis induced by H_2_O_2_ but not LPS.

### MiR-181c agomir accelerates apoptosis of neuronal cells co-cultured with microglial cells

**Figure 5.**
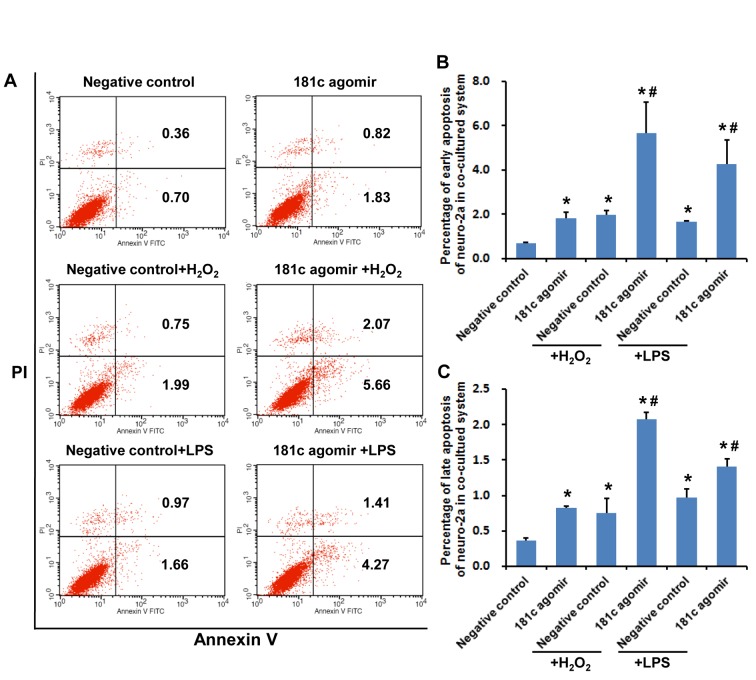
**MiR-181c agomir induces apoptosis of Neuro-2a cells co-cultured with BV2 microglial cells upon oxidative stress and inflammation. A**) Apoptotic cells were detected by flow cytometry. Second and fourth quadrants represent late and early apoptotic fractions, respectively. **B**) Early apoptosis of Neuro-2a cells. **C**) Late apoptosis of Neuro-2a cells. *P < 0.05 vs. negative control group; ^#^*P* < 0.05 vs. negative control + H_2_O_2_ or negative control + LPS group.

We further examined the role of miR-181c in oxidative stress- or inflammation-induced apoptosis using an established BV2-Neuro-2a cell co-culture system, with apoptotic cells detected by flow cytometry (Fig. 5A). Similar to the results obtained for Neuro-2a cells cultured alone, apoptosis was increased in the presence of H_2_O_2_ and LPS in Neuro-2a cells cultured with BV2 microglial cells; however, the apoptotic fraction was increased in cells treated with miR-181c agomir and H_2_O_2_ as well as with LPS (Fig. 5B, C, *P* < 0.05), indicating that LPS had a greater effect on Neuro-2a cells co-cultured with BV2 cells as compared with those cultured alone.

### MiR-181c agomir promotes brain ischemia-reperfusion injury in a mouse MCAO model

**Figure 6.**
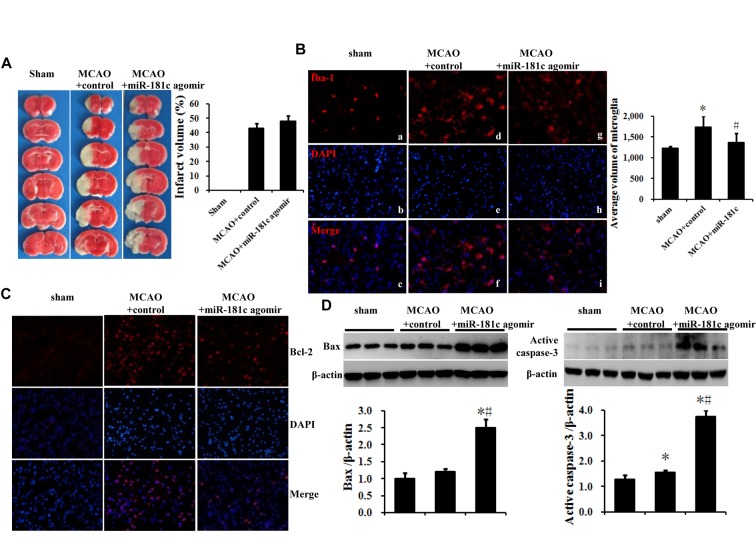
**MiR-181c agomir exacerbates brain ischemia-reperfusion injury in an MCAO mouse model. A**) Cerebral infarct volume was evaluated by TTC staining of coronal brain sections. **B**) MiR-181c agomir inhibited microglia activation in the ipsilateral cortex, as determined by immunocytochemistry. **C**) MiR-181c agomir inhibited the expression of the anti-apoptotic protein Bcl-2 in the ipsilateral cortex, as detected by immunocytochemistry. **D**) MiR-181c agomir increased the levels of the pro-apoptotic factors Bax and activated caspase-3 in the ipsilateral cortex, as detected by western blotting. Values represent mean ± SEM. *P < 0.05 vs. sham group, ^#^*P* < 0.05 vs. MCAO + control group.

We assessed the function of miR-181c *in vivo* using a mouse model of focal cerebral ischemia. Cerebral infarct volume was measured 72 h after reperfusion, and the expressions of the microglia marker Iba-1, the miR-181c target Bcl-2, and the pro-apoptotic factors Bax and activated caspase-3 in the ipsilateral cortex were detected by immunocytochemistry and western blotting. Intracerebroventricular injection of miR-181c agomir increased cerebral infarct volume as compared with control MCAO mice, but this change was not statistically significant (Fig. 6A). In sham-operated animals, ramified Iba-1^+^ microglia (known as resting microglia) were uniformly distributed throughout the cortex and exhibited a small cytoplasmic volume (Fig. 6B, a-c). In the MCAO group, most Iba-1^+^ microglia were hypertrophied, with dense cytoplasm and rounded cell bodies (Fig. 6B, d-f); this phenotype was abolished by miR-181c agomir treatment (Fig. 6B, g-i). The number of Bcl-2^+^ cells was increased in control MCAO mice, which was abrogated by miR-181c agomir treatment (Fig. 6C). Furthermore, the upregulation of Bax and activated caspase-3 levels in the control MCAO group was potentiated by miR-181c agomir treatment (Fig. 6D; *P* < 0.05). These results indicate that miR-181c suppresses microglia activation and the expression of the anti-apoptotic factor Bcl-2 while stimulating pro-apoptotic factors Bax and activated caspase-3.

## DISCUSSION

The present study investigated the clinical relevance of miR-181c in acute stroke patients, and examined miR-181c function in an acute stroke model and in BV2 and Neuro-2a cells under conditions of oxidative stress and inflammation. Our results reveal a pro-apoptotic function of miR-181c both *in vitro* and *in vivo*.

We found that hsa-miR-181c levels were downregulated in the lymphocytes and plasma of stroke patients. This is consistent with a previous observation that miR-181 levels were reduced in the brain tissue of rats subjected to transient focal ischemia [12], and were decreased in the ischemic penumbra, but increased in the ischemic core following transient focal ischemia in the mouse [13]. Our analysis revealed a positive correlation between NIHSS score and miR-181c level although this was not statistically significant, suggesting that miR-181c is a potential risk factor for stroke. MiR-181 family members play a key role in the regulation of lymphocyte development and function [6-11]; in particular, miR-181c has been shown to suppress the activation of CD4+ T cells [10]. Our data demonstrated that miR-181c expression was positively and negatively correlated with neutrophil and lymphocyte percentage, respectively. Given that higher neutrophil numbers before thrombolysis for cerebral ischemia predict worse outcomes^20^ as well as the association between neutrophil-to-lymphocyte ratio and risk of stroke in patients with atrial fibrillation [21] we suggest that miR-181c is an immune regulator that modulates the neutrophil-to-lymphocyte ratio following acute ischemic stroke.

To clarify the precise function of miR-181c, we evaluated the role of miR-181c in the apoptosis of BV2 and Neuro-2a cells cultured alone or together with H_2_O_2_ or LPS treatment. MiR-181 has been shown to inhibit inflammation in astrocytes, microglia, and dendritic cells by suppressing cytokine levels [11,14,15,22], and miR-181c-transfected BV2 cell culture medium reduced neuronal apoptosis induced by LPS [15]. However, these studies did not examine the link between inhibition of inflammation and immune cell apoptosis. In the present study, we showed that the miR-181c agomir suppressed proliferation and induced apoptosis of BV2 microglial cells cultured alone with or without H_2_O_2_/LPS treatment. A previous study found that miR-181a prevented Neuro-2a cell death induced by serum deprivation or H_2_O_2_.^14^ We observed that in Neuro-2a cells cultured alone, miR-181c agomir accelerated apoptosis induced by H_2_O_2_, but not by LPS; however, when these cells were co-cultured with BV2 microglial cells, the miR-181c agomir accelerated both H_2_O_2_- or LPS-induced Neuro-2a apoptosis. The difference between our results and the previous study [15] can be explained by the fact that there is a bi-directional interaction between Neuro-2a and BV2 cells in the co-culture system, as opposed to culturing in conditioned medium where the interactions are unilateral.

In our ischemic stroke model, we demonstrated that the miR-181c agomir exacerbated brain ischemia-reperfusion injury, consistent with the reported effects of miR-181a in a mouse stroke model [13]. Bcl-2, a mitochondrial membrane-associated protein, is a target of miR-181 that mediates miR-181a-mediated Neuro-2a cell death upon oxidative stress [14], miR-181a-triggered mitochondrial dysfunction and astrocyte death upon glucose deprivation [9], and miR-181d-induced glioma cell apoptosis [23]. MiR-181c was also shown to increase the production of reactive oxygen species by targeting cyclooxygenase 1, a subunit of mitochondrial complex IV of the electron transport chain [24]. Hence, miR-181 causes cell injury mainly by targeting mitochondrial proteins. Indeed, our *in vitro* study showed that miR-181c increased the apoptosis of Neuro-2a cells to a greater extent under conditions of oxidative stress than under inflammation, an effect that may be exerted via activation of a mitochondria-dependent apoptosis pathway. In MCAO mice, miR-181c modestly increased infarct volume, which was associated with downregulation of the anti-apoptotic protein Bcl-2 and upregulation of the pro-apoptotic factors, Bax and activated caspase-3.

We found that miR-181c was involved in acute ischemic stroke, promoting apoptosis of BV2 and Neuro-2a cells and aggravating brain ischemia-reperfusion injury in a mouse model of stroke via modulation of pro- and anti-apoptotic proteins. These results suggest that high miR-181c level is a potential risk factor for ischemic stroke and apoptosis of various types of neural cells during the acute stage. Future studies will focus on the therapeutic potential of miR-181c delivered via intravenous injection in the treatment of ischemic stroke.

## References

[1] Lozano R, Naghavi M, Foreman K, Lim S, Shibuya K, Aboyans V, et al (2012). Global and regional mortality from 235 causes of death for 20 age groups in 1990 and 2010: a systematic analysis for the Global Burden of Disease Study 2010. Lancet, 380: 2095-2128. 23245604 10.1016/S0140-6736(12)61728-0 PMC10790329

[2] Jickling GC, Sharp FR (2011). Blood biomarkers of ischemic stroke. Neurotherapeutics, 8: 349-360. 21671123 10.1007/s13311-011-0050-4 PMC3250275

[3] Suzumura A (2013). Neuron-microglia interaction in neuroinflammation. Curr Protein Pept Sci, 14:16-20. 23544747 10.2174/1389203711314010004

[4] Lagos-Quintana M, Rauhut R, Lendeckel W, Tuschl T (2001). Identification of novel genes coding for small expressed RNAs. Science, 294: 853-858. 11679670 10.1126/science.1064921

[5] Chen CZ, Li L, Lodish HF, Bartel DP (2004). MicroRNAs modulate hematopoietic lineage differentiation. Science, 303: 83-86. 14657504 10.1126/science.1091903

[6] Zhang Q, Sun H, Jiang Y, Ding L, Wu S, Fang T, et al (2013). MicroRNA-181a suppresses mouse granulosa cell proliferation by targeting activin receptor IIA. PLoS One, 8: e59667. 23527246 10.1371/journal.pone.0059667 PMC3604175

[7] Shi L, Cheng Z, Zhang J, Li R, Zhao P, Fu Z, et al (2008). hsa-mir-181a and hsa-mir-181b function as tumor suppressors in human glioma cells. Brain Res, 1236: 185-193. 18710654 10.1016/j.brainres.2008.07.085

[8] Xu Z, Jiang J, Xu C, Wang Y, Sun L, Guo X, et al (2013). MicroRNA-181 regulates CARM1 and histone arginine methylation to promote differentiation of human embryonic stem cells. PLoS One, 8: e53146. 23301034 10.1371/journal.pone.0053146 PMC3536801

[9] Ouyang YB, Lu Y, Yue S, Giffard RG (2012). miR-181 targets multiple Bcl-2 family members and influences apoptosis and mitochondrial function in astrocytes. Mitochondrion, 12: 213-219. 21958558 10.1016/j.mito.2011.09.001 PMC3250561

[10] Xue Q, Guo ZY, Li W, Wen WH, Meng YL, Jia LT, et al (2011). Human activated CD4(+) T lymphocytes increase IL-2 expression by downregulating microRNA-181c. Mol Immunol, 48: 592-599. 21112091 10.1016/j.molimm.2010.10.021

[11] Hutchison ER, Kawamoto EM, Taub DD, Lal A, Abdelmohsen K, Zhang Y, et al (2013). Evidence for miR-181 involvement in neuroinflammatory responses of astrocytes. Glia, 61: 1018-1028. 23650073 10.1002/glia.22483 PMC4624280

[12] Jeyaseelan K, Lim KY, Armugam A (2008). MicroRNA expression in the blood and brain of rats subjected to transient focal ischemia by middle cerebral artery occlusion. Stroke, 39: 959-966. 18258830 10.1161/STROKEAHA.107.500736

[13] Ouyang YB, Lu Y, Yue S, Xu LJ, Xiong XX, White RE, et al (2012). miR-181 regulates GRP78 and influences outcome from cerebral ischemia in vitro and in vivo. Neurobiol Dis, 45: 555-563. 21983159 10.1016/j.nbd.2011.09.012 PMC3251314

[14] Moon JM, Xu L, Giffard RG (2013). Inhibition of microRNA-181 reduces forebrain ischemia-induced neuronal loss. J Cereb Blood Flow Metab, 33:1976-1982. 24002437 10.1038/jcbfm.2013.157 PMC3851907

[15] Zhang L, Dong LY, Li YJ, Hong Z, Wei WS (2012). The microRNA miR-181c controls microglia-mediated neuronal apoptosis by suppressing tumor necrosis factor. J Neuroinflammation, 9: 211. 22950459 10.1186/1742-2094-9-211 PMC3488569

[16] Zhang L, Li YJ, Wu XY, Hong Z, Wei WS (2015). MicroRNA-181c negatively regulates the inflammatory response in oxygen-glucose-deprived microglia by targeting Toll-like receptor 4. J Neurochem, 132:713-723. 25545945 10.1111/jnc.13021

[17] Zhao H, Wang J, Gao L, Wang R, Liu X, Gao Z, et al (2013). MiRNA-424 protects against permanent focal cerebral ischemia injury in mice involving suppressing microglia activation. Stroke, 44:1706-1713. 23613494 10.1161/STROKEAHA.111.000504

[18] Liu P, Zhao H, Wang R, Wang P, Tao Z, Gao L, et al (2015). MicroRNA-424 protects against focal cerebral ischemia and reperfusion injury in mice by suppressing oxidative stress. Stroke, 46:513-519. 25523055 10.1161/STROKEAHA.114.007482

[19] Yin XM, Luo Y, Cao G, Bai L, Pei W, Kuharsky DK, et al (2002). Bid-mediated mitochondrial pathway is critical to ischemic neuronal apoptosis and focal cerebral ischemia. J Biol Chem, 44: 42074-81. 10.1074/jbc.M204991200 12200426

[20] Maestrini I, Strbian D, Gautier S, Haapaniemi E, Moulin S, Sairanen T, et al (2015). Higher neutrophil counts before thrombolysis for cerebral ischemia predict worse outcomes. Neurology, 85:1408-1416. 26362283 10.1212/WNL.0000000000002029 PMC4626239

[21] Hyun S, Kwon S, Cho S, Park S, Jung W, Moon S, et al (2015). Can the Neutrophil-to-Lymphocyte Ratio Appropriately Predict Carotid Artery Stenosis in Patients with Ischemic Stroke?-A Retrospective Study. J Stroke Cerebrovasc Dis, 24:2646-2651. 26316355 10.1016/j.jstrokecerebrovasdis.2015.07.024

[22] Wu C, Gong Y, Yuan J, Zhang W, Zhao G, Li H, et al (2012). microRNA-181a represses ox-LDL-treated inflammatory response in dendritic cell by targeting c-Fos. J Lipid Res, 53: 2355-2363. 22956783 10.1194/jlr.M028878 PMC3466004

[23] Wang XF, Shi ZM, Wang XR, Cao L, Wang YY, Zhang JX, et al (2012). MiR-181d acts as a tumor suppressor in glioma by targeting K-ras and Bcl-2. J Cancer Res Clin Oncol, 138: 573-584. 22207524 10.1007/s00432-011-1114-x PMC11824363

[24] Das S, Ferlito M, Kent OA, Fox-Talbot K, Wang R, Liu D, et al (2012). Nuclear miRNA regulates the mitochondrial genome in the heart. Circ Res, 110:1596-1603. 22518031 10.1161/CIRCRESAHA.112.267732 PMC3390752

